# The effect of nasal breathing on the exercise induced bronchospasm in children with allergic asthma and rhinitis

**DOI:** 10.1186/2045-7022-3-S2-P7

**Published:** 2013-07-16

**Authors:** Mirjana Turkalj, Martina Canaki, Robert Magdic, Marcel Lipej, Sandra Bulat, Jelena Zivkovic, Davor Plavec

**Affiliations:** 1 Children's Hospital Srebrnjak, Zagreb, Croatia; 2 Children's Hospital Srebrnjak, Allergy Department, Zagreb, Croatia

## Background

Allergic rhinitis (AR) is very common in children and it affects 10--40% of children world-wide. Asthma and AR commonly coexist and we can find up to 30% of asthma in patients with AR and up to 90% of AR in patients with asthma. Moreover, these two disorders seem to influence each other's activity and intensity. Physical activity is commonly prescribed as a rehabilitation treatment for asthma in children although there is a high percentage of asthmatics with exercise-induced bronchospasm (EIB) at that age and recent studies show beneficial effects of aerobic training on allergic inflammation. EIB can be one of the reasons for low adherence to physical training. The aim of this study was to test if a nose clip during a 6 minute free running test changes the magnitude of EIB according to the severity of AR.

## Methods

The study was conducted in 55 children (24 girls, mean age 12.6 yrs) with moderate persistent asthma and AR in an Asthma Camp, at island Lošinj. Their asthma was controlled under their regular treatment and they were daily participating in an aerobic fitness program. They were divided in two subgroups according to the median of intensity of their nasal symptoms (less nasal symptoms -- LNS; more nasal symptoms -- MNS). Spirometry was performed before, 3' and 20' after 2 exercises (6 minutes free running with and without a nose clip) done a day apart.

## Results

Two subgroups (LNS and MNS) were not significantly different according to their demographic characteristics, sensitization profile, asthma control, lung function and exhaled NO measurements and physical fitness (p>0.05 for all). There was a significantly greater fall in FEV1 3' after exercise with a nose clip in the LNS subgroup than in the MNS subgroup (p<0.001) and compared to testing without the nose clip (LNS, p=0.009; MNS, p=0.010). Contrary to the testing with the nose clip there were no significant differences in the FEV1 fall after exercise when the same patients were tested without the nose clip during exercise (p>0.68).

## Conclusion

It seems that regular mouth breathing due to nasal congestion somehow protects patients with asthma and AR from EIB.

